# Interleukin-1β pre-treated bone marrow stromal cells alleviate neuropathic pain through CCL7-mediated inhibition of microglial activation in the spinal cord

**DOI:** 10.1038/srep42260

**Published:** 2017-02-14

**Authors:** Jian Li, Guoying Deng, Haowei Wang, Mei Yang, Rui Yang, Xiangnan Li, Xiaoping Zhang, Hongbin Yuan

**Affiliations:** 1 Department of Anesthesiology, Changzheng Hospital, Second Military Medical University, 415 Fengyang Road, Shanghai, 200003, China; 2 Trauma Center, Shanghai General Hospital, Shanghai Jiaotong University School of Medicine, 650 Xin Songjiang Road, Shanghai, 201620, China; 3 Department of Anesthesiology, the Third People’s Hospital of Yancheng, Yancheng, 224001, China; 4 Department of Interventional & Vascular Surgery, Tongji University School of Medicine, Shanghai 200072, China

## Abstract

Although neuropathic pain is one of the most intractable diseases, recent studies indicate that systemic or local injection of bone marrow stromal cells (BMSCs) decreases pro-inflammatory cytokines release and alleviates neuropathic pain. However, it is still not clear whether pre-treated BMSCs have a strong anti-inflammatory and/or analgesia effect. Using the spinal nerve ligation model of neuropathic pain, IL-1β pre-treated BMSCs (IL-1β-BMSCs) were injected into rats followed by SNL in order to determine possible effects. Results indicated that IL-1β-BMSCs were more efficacious in both amelioration of neuropathic pain and inhibition of microglia activation. Specifically, microglia inhibition was found to be mediated by chemokine C-C motif ligand 7 (CCL7) but not CCL2. Results also showed that IL-1β-BMSCs had a stronger inhibitory effect on astrocyte activation as well as CCL7 release, which was found to be mediated by IL-10 not transforming growth factor-β1. In addition, we also found directional migration of IL-1β-BMSCs was mediated by inceased C-X-C motif chemokine ligand (CXCL) 13 expression following SNL. In conclusion, our results indicated IL-1β-BMSCs could inhibit microglia activation and neuropathic pain by decreasing CCL7 level in spinal cord.

Neuropathic pain arises from lesions or dysfunctions of the nervous system and is one of the most refractory human diseases^1^, with allodynia and hyperalgesia comprising the two major symptoms associated with this pain^2^. The mechanisms of neuropathic pain are very complex and involve both structural and functional changes throughout the nociceptive pathway, from the site of peripheral nerve injury, to dorsal root ganglion (DRG), as well as spinal cord and brain^3^,^4^. Increasing evidence indicates that neuroinflammation and the immune system play an important role in the occurrence and development of neuropathic pain^5^,^6^,^7^. Several animal models of neuropathic pain have been developed, but L5 spinal nerve ligation (SNL) model has been one of the most widely used models^8^,^9^,^10^.

Glia, specifically microglia have emerged as key contributors to pathological and chronic pain mechanisms^11^. Microglia are immune cells in the central nervous system (CNS) and play a key role in the development and homeostasis of the CNS^12^ and which are known to be activated during neuropathic pain. Activated microglia release proinflammatory cytokines and chemokines, which are known to modulate the pain process^13^. These glia can be activated through a variety of traumas including peripheral nerve injury and CNS injury, whereby dysregulation of microglia activation contributes to hypersensitivity associated with neuropathic pain^10^. Activated microglia can also initiate a series of signaling cascades, which are known to enhance neuronal excitability and synaptic plasticity, thereby facilitating neuropathic pain^13^. In addition, a recent study showed that microglia can act synergistically with peripheral monocytes to initiate hypersensitivity after peripheral nerve injury, and therefore contribute to the transition from acute to chronic pain^14^.

Bone marrow stromal cells (BMSCs) are a population of progenitor cells of mesodermal origin that are present in the bone marrow of adults and give rise to various tissues throughout the body^15^. BMSCs have emerged as a novel candidate for cell-based therapies, with a diverse spectrum of potential to prevent and treat clinical diseases^16^. BMSCs are known to elicit anti-inflammatory and neuroprotective effects and have been applied in the treatment of a variety of inflammatory diseases^17^,^18^. Furthermore, these cells can decrease the release of pro-inflammatory cytokines and chemokines and reduce pro-inflammatory cell migration into sites of injury^16^. A recent study also suggested that factors released by BMSCs can modulate the activation of microglia^19^. In addition, transplantation of BMSCs can positively alter the spinal microenvironment and contribute to glial repair after spinal cord injury^20^. Recently, a study indicated that intrathecal injection of BMSCs inhibited pro-inflammatory cytokines release and alleviated neuropathic pain via transforming growth factor-β (TGF-β) secretion^5^, however, it is still not clear whether cytokines pre-treatment can enhance the inhibitory effect of BMSCs on pro-inflammatory cytokines release and neuropathic pain. As IL-1β is one of the most common pro-inflammatory cytokines, we decided to use IL-1β to pre-treat BMSCs to acquire IL-1β-BMSCs and further test the action of it on neuropathic pain.

Chemokines are a family of small secreted proteins and are well known regulators of peripheral immune cell trafficking^21^. Increasing evidence indicates that chemokines play an important role in neuroinflammation and neurodegeneration^22^,^23^ and several chemokines (e.g., chemokines C-C motif ligand 2 (CCL2), 7(CCL7) and C-X-C motif chemokine ligand13 (CXCL13)) have been shown to mediate neuronal-glial interactions in the CNS and contribute to both neuropathic and inflammatory pain^21^,^24^. CCL2 and CCL7 are two common activators of spinal microglia in neuropathic pain conditions^24^,^25^ that promote recruitment of microglia and macrophages in the development of multiple sclerosis lesions^26^. CCL7 mainly expresses in astrocytes after partial sciatic ligation, and can activate microglia through C-C chemokine receptor type 2 (CCR2), moreover IL-6 can activate astrocytes to express CCL7^24^. While CXCL13 is a chemokine that is upregulated in brain and spinal cord under pathological conditions, such as neuroborreliosis, autoimmune demyelination, and primary CNS lymphoma^27^,^28^. In the spinal cord, CXCL13 derived from neurons activates astrocytes via C-X-C chemokine receptor type 5 (CXCR5) to facilitate neuropathic pain^8^. In addition, a recent study showed that the CXCL12-CXCR4 pathway mediated the directional migration of BMSCs after chronic constriction injury (CCI) in mice^5^, however, it remains unclear whether CXCL13 can mediates BMSCs migration.

In order to test whether IL-1β-BMSCs had a better analgesic effect than BMSCs, withdrawal threshold and withdrawal latency were used to represent mechanical allodynia and thermal hyperalgesia, respectively. Using intrathecal (i.t.) administration of IL-1β-BMSCs or BMSCs (2.5 × 10^6^ cells/rat) in the early phase (initial phase) and the late phase (maintenance phase) of neuropathic pain, we found that IL-1β-BMSCs administration more effectively alleviated neuropathic pain after SNL compared to BMSCs administration alone. To further explore the underlying mechanisms by which IL-1β-BMSCs produced a better analgesic effect, microglia activation and proinflammatory cytokines level were detected. We found that IL-1β-BMSCs had a stronger inhibitory effect both on activation of microglia and on secretion of proinflammatory factors. This effect is at least partly mediated by CCL7 pathway, because IL-1β-BMSCs significantly decreased CCL7 level in spinal cord, and intrathecal injection of exogenous CCL7 reversed the analgesic and microglia inhibition effect of IL-1β-BMSCs. CCL7 mainly expressed in astrocytes after nerve ligation^24^, our results showed that IL-1β-BMSCs treatment inhibited astrocyte activation and CCL7 expression through release of IL-10 both *in vitro* and *in vivo*. What’s more, we also found IL-1β-BMSCs selectively targeted ipsilateral spinal cord, which was mediated through a C-X-C motif chemokine ligand 13 (CXCL13) mediaged pathway in spinal cord after SNL. Finally IL-1β pretreatment significantly increased the number of BMSCs migrated to CXCL13 *in vitro*, and *in vivo* konck down of CXCL13 in spinal cord using shRNA significantly decreased the number of BMSCs on the surface of ipsilateral spinal cord, but the specific mechanisms need further studies. In conclusion, our results indicated intrathecal injection of IL-1β-BMSCs could inhibit microglia activation and alleviate neuropathic pain through decreasing CCL7 level in spinal cord.

## Results

### BMSCs pre-treated with different concentrations of IL-1β alleviated neuropathic pain in a concentration dependent manner

**Figure 1:**
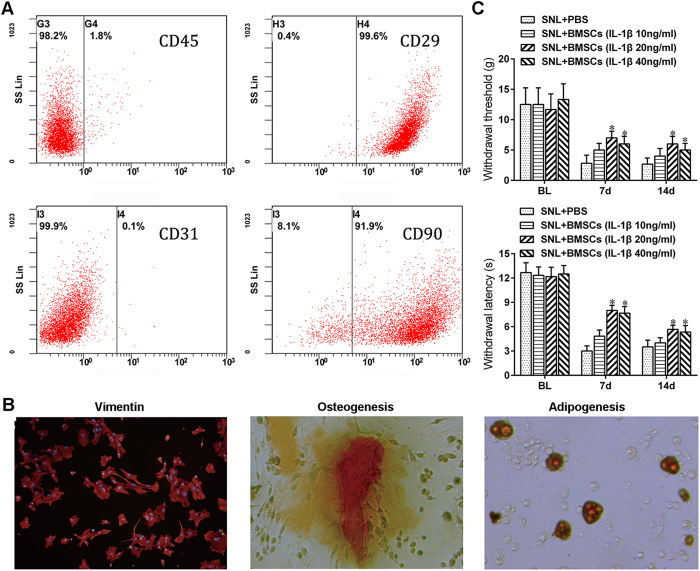
BMSCs pre-treated with different concentrations of IL-1β alleviated neuropathic pain in a concentration dependent manner. (**A**) Flow cytometry analysis of isolated BMSCs. More than 90% of BMSCs were CD29 (+) and CD90 (+), and less than 2% of BMSCs were CD45 (+) or CD31 (+). (**B**) BMSCs were positive for vimentin (V9) and had osteogenesis and adipogenesis potential. (**C**) Inhibition of mechanical allodynia and thermal hyperalgesia by i.t. injection of BMSCs pre-treated with different concentrations of IL-1β. *P < 0.05, compared with PBS group; n = 6 cultures/group for cell experience; 24 rats in total for behavioral test and n = 6 rats/group. Statistical significance was determined using a two-way repeated-measures ANOVA followed by Bonferroni’s post-hoc test. All data are expressed as the mean ± SEM.

We isolated BMSCs from Sprague-Dawley rats transfected with green fluorescent protein (GFP), and then cultured these cells in complete medium. Following the 3^rd^ passage, we used flow cytometric analysis to identify BMSCs using various clusters of differentiation proteins (CD) including CD90, CD45, CD29, and CD31^19^ with results indicating that more than 90% of the cells were positive for CD29 and CD90, whereas less than 2% were positive for CD31 or CD45 (Fig. 1A). Immunofluorescent staining showed that the BMSCs were positive for vimentin (V9) and had osteogenesis and adipogenesis potential (Fig. 1B). To test the optimum concentration of IL-1β to pre-treat BMSCs, we divided the BMSCs at the 4^th^ passage into four groups, which were then stimulated with IL-1β at the following concentrations: 0, 10, 20, and 40 ng/ml, respectively, for 24 h. We found that BMSCs pre-treated with 20 ng/ml or 40 ng/ml of IL-1β significantly alleviated mechanical allodynia and thermal hyperalgesia, and moreover, BMSCs pre-treated with 20 ng/ml of IL-1β achieved the most powerful analgesic effect (Fig. 1C).

### Early or late treatment with IL-1β-BMSCs was more effective than the BMSCs treatment alone for relief of neuropathic pain after SNL

**Figure 2:**
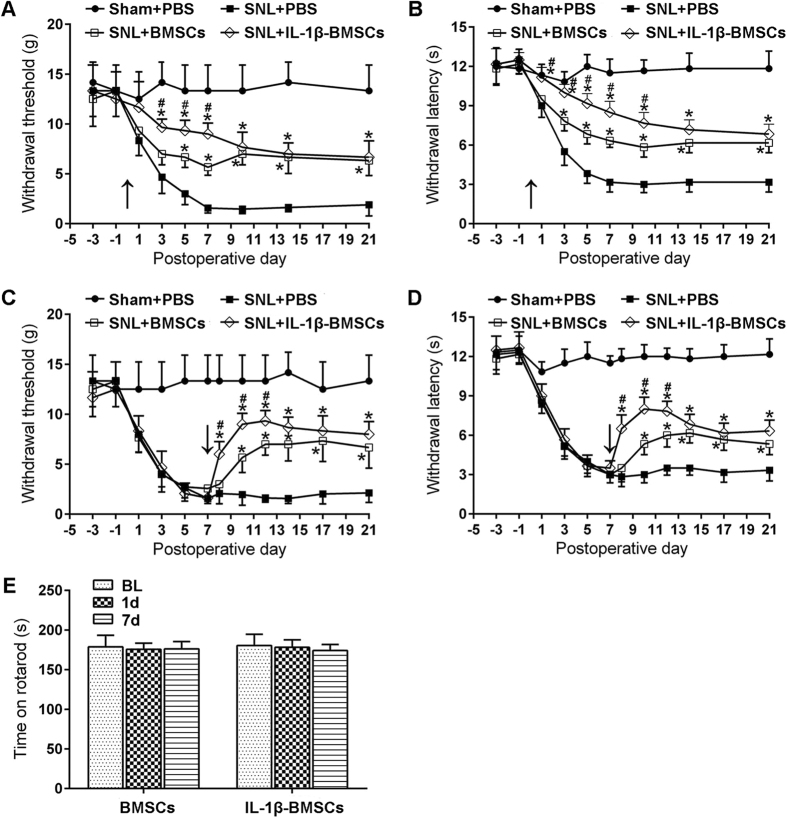
Stronger inhibition of SNL-induced neuropathic pain in rats by injection of IL-1β-BMSCs compared to BMSCs alone. (**A** and **B**) Inhibition of (**A**) mechanical allodynia and (**B**) thermal hyperalgesia for three weeks following early treatment (given 0.5 h before SNL) with IL-1β-BMSCs or BMSCs, as well as faster and stronger inhibition of neuropathic pain by IL-1β-BMSCs compared to BMSCs for the first seven days. (**C** and **D**) BMSCs or IL-1β-BMSCs delivered on day 7 after SNL reversed (**C**) mechanical allodynia and (**D**) thermal hyperalgesia for at least two weeks, with IL-1β-BMSCs producing a faster and stronger reversal than BMSCs. (**E**) Both IL-1β-BMSCs and BMSCs did not affect motor function as evaluated by the rotarod test. Arrows in (**A–D**) indicate the time points of IL-1β-BMSCs or BMSCs injection. *P < 0.05, compared with PBS, ^#^P < 0.05; 36 rats in total and n = 6 rats/group. Statistical significance was determined by two-way repeated-measures ANOVA followed by Bonferroni’s post-hoc test. All data are expressed as the mean ± SEM.

We induced neuropathic pain in rats via an L5 SNL, a classical neuropathic pain model^8^,^29^. To test the hypothesis that IL-1β-BMSCs treatment was more effective than BMSCs treatment alone in alleviating neuropathic pain, we administrated an i.t. injection of IL-1β-BMSCs or BMSCs (2.5 × 10^6^) with a purity of over 90% (Fig. 1A) into spinal cerebrospinal fluid via lumbar puncture, just prior to SNL. As shown in Fig. 2A and B, i.t. injection of IL-1β-BMSCs produced a marked inhibition of SNL-induced mechanical allodynia and thermal hyperalgesia beginning on day 3 post-SNL and lasted for more than two weeks. In addition, the antinociceptive effects of IL-1β-BMSCs was more significant than BMSCs treatment alone (Fig. 2A and B). To examine the effects of IL-1β-BMSCs treatment on the maintenance of neuropathic pain, we administered either IL-1β-BMSCs or BMSCs on day 7 post-SNL, and as shown in Fig. 2C and D, IL-1β-BMSCs effectively reversed mechanical allodynia and thermal hyperalgesia within one day after injection, which lasted for more than 2 weeks. Similar to the previous results, the antinociceptive effects of IL-1β-BMSCs treatment was significantly stronger than BMSCs treatment alone during the first 5 days after injection (Fig. 2C and D). Neither BMSCs treatment alone nor IL-1β-BMSCs treatment affected motor function, as evaluated by a rotarod test (Fig. 2E).

### IL-1β-BMSCs treatment resulted in stronger neuroinflammation inhibition

**Figure 3:**
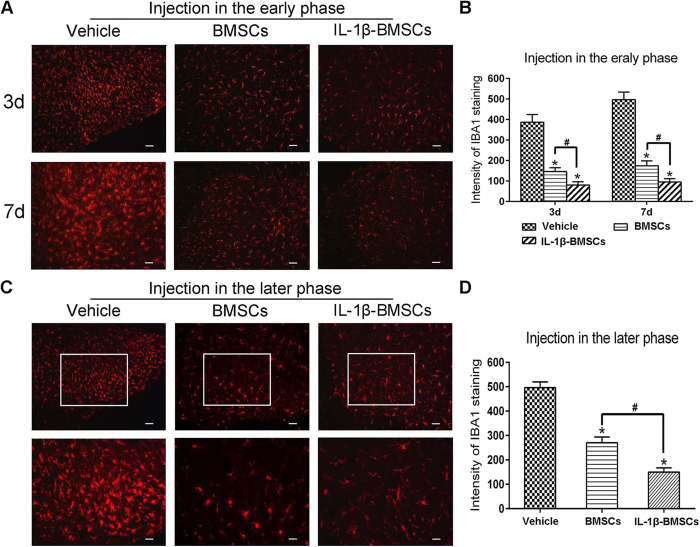
IL-1β-BMSCs i.t. injected inhibited SNL-induced activation of microglia. (**A** and **B**) Inhibition of SNL-induce microglia activation by early injection (0.5 h prior to SNL) of IL-1β-BMSCs or BMSCs with a stronger inhibition produced by i.t. injection of IL-1β-BMSCs compared with i.t. BMSCs. Upper panels were spinal cords on day 3 after SNL, and lower panels were spinal cord on day 7. Scale bars = 100 μm. (**C** and **D**) Inhibition of microglia activation by later injection (day 7 after SNL) of IL-1β-BMSCs or BMSCs. IL-1β-BMSCs further decreased microglia activation compared with BMSCs. Lower panels were the amplification of the upper rectangular area. Scale bars: 100 μm (top panels) and 50 μm (bottom panels). *P < 0.05 compared with vesicle, ^#^P < 0.05; 24 rats in total and n = 8 rats/group (4 rats for early injection, and 3–4 rats for later phase). Statistical significance was determined by one-way ANOVA followed by Bonferroni’s post-hoc test. All data are expressed as the mean ± SEM.

To examine the effects of IL-1β-BMSCs treatment on SNL-induced neuroinflammation, we tested the activation of microglia in the dorsal horn of spinal cord. As shown in Fig. 3A and B, injection of IL-1β-BMSCs in the early phase (just before SNL) significantly decreased the activation of microglia induced by SNL on day 3 and day 7 (Fig. 3A and B). We further examined the effects of IL-1β-BMSCs treatment on activated microglia at the later phase (IL-1β-BMSCs injected on day 7 after SNL). Three days after injection, L5 spinal cord segments were collected and stained with ionized calcium binding adaptor molecule 1(IBA1), a marker for activated microglia. As shown in Fig. 3C and D, the intensity of IBA1 significantly decreased in both IL-1β-BMSCs treated and BMSCs treated groups compared with the vehicle group (Fig. 3C and D). Injection IL-1β-BMSCs at both the early and late phases produced a stronger inhibition of microglial activation compared to injection of BMSCs alone.

**Figure 4:**
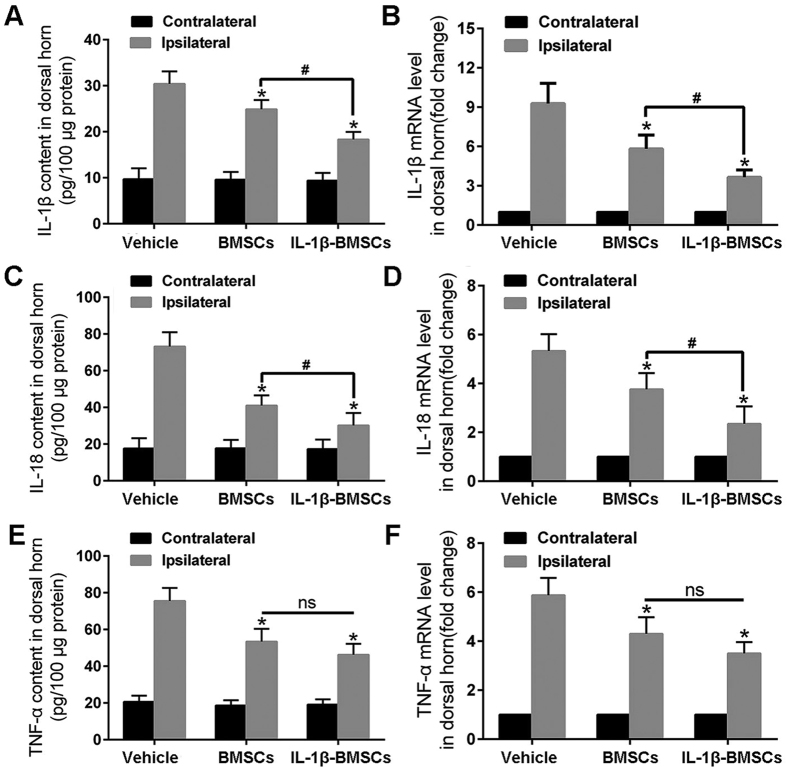
IL-1β-BMSCs decreased both protein and mRNA levels of proinflammatory cytokines. (**A**–**F**) Protein and mRNA levels of proinflammatory cytokines in the dorsal horn of the spinal cord, including (**A** and **B**) IL-1β, (**C** and **D**) IL-18, and (**E** and **F**) TNF-α. IL-1β-BMSCs decreased (**A**–**D**) IL-1β and IL-18 levels, but not TNF-α levels, compared with (**E** and **F**) BMSCs. *P < 0.05, compared with vehicle control, ^#^P < 0.05; 24 rats in total and n = 8 rats/group (4 rats for ELISA and another 4 rats for q-PCR). One-way ANOVA followed by Bonferroni’s post-hoc test was used and all data expressed as the mean ± SEM. Ns: no significance.

To further examine changes in neuroinflammation, we detected the levels of several of the most common proinflammatory cytokines including IL-1β and tumor necrosis factor-α (TNF-α), as well as IL-18, which has been found to be closely related with neuropathic pain^30^,^31^,^32^. ELISA and quantitative reverse transcriptase-PCR (qRT-PCR) were used to detect protein and mRNA levels, respectively. BMSCs or IL-1β-BMSCs were injected into the dorsal horn of the spinal cord immediately prior to SNL and the dorsal horn was collected on day 7. Although both IL-1β-BMSCs and BMSCs treatments decreased the levels of proinflammatory cytokines, IL-1β-BMSCs treatment produced larger decreases in the levels of IL-1β and IL-18, but not TNF-α, compared to BMSCs treatment (Fig. 4).

### CCL7 effectively reversed the antinociceptive effect of IL-1β-BMSCs treatment

**Figure 5:**
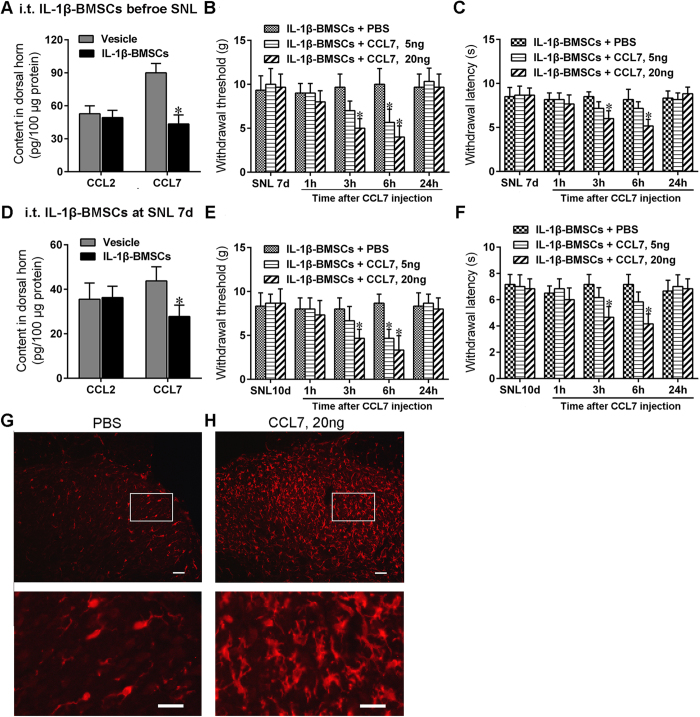
CCL7 reversed the antiallodynic effect of IL-1β-BMSCs. (**A**) ELISA analysis showed that CCL7 levels in the dorsal horn decreased following IL-1β-BMSCs injection (0.5 h before SNL; tissue collected on day 7 after SNL). (**B** and **C**) Both high (20 ng) and low (5 ng) levels of CCL7 on day 7 after SNL (and therefore IL-1β-BMSCs injection), decreased the (**B**) withdrawal threshold and (**C**) withdrawal latency. (**D**) IL-1β-BMSCs injected on day 7 after SNL, decreased CCL7 levels in the dorsal horn (tissue collected on day 10 after SNL (day 3 after IL-1β-BMSCs injection)). (**E** and **F**) High levels of CCL7 (20 ng), but not lower levels (5 ng), injected on day 10 after SNL (day 3 after IL-1β-BMSCs injection) reversed the antiallodynic effects of IL-1β-BMSCs. (**G** and **H**) Microglia in the dorsal horn were activated by injection of **(H)** high levels of CCL7on day 7 after SNL compared to **(G)** vehicle group (day 7 after IL-1β-BMSCs injection). Scale bars = 100 μm (top panels) and 50 μm (bottom panels). *P < 0.05 compared with PBS, ^#^P < 0.05 compared with vehicle; 72 rats in total and n = 6 rats/group. Statistical significance was determined by (**A** and **D**) student’s t test, or (**B**,**C**,**E** and **F**) two-way repeated-measures ANOVA followed by Bonferroni’s post-hoc test. All data were expressed as the mean ± SEM.

CCL7 and CCL2 are common chemotactic factors that mediate neuronal-glia interactions in the CNS and contribute to the development of neuropathic pain^13^. To test the hypothesis that injection of IL-1β-BMSCs alleviates neuropathic pain through a chemokine axis, we detected changes in the levels of CCL7 and CCL2 in the dorsal horn of spinal cord after SNL. Early treatment with IL-1β-BMSCs, given just before SNL, inhibited SNL-induced CCL7 rather than CCL2 expression in the dorsal horn of spinal cord (Fig. 5A). We then evaluated the effects of exogenous recombinant CCL7 on the antinociceptive effect of IL-1β-BMSCs treatment after SNL. We tested the withdrawal threshold and withdrawal latency to evaluate mechanical allodynia and thermal hyperalgesia, respectively. Two doses of CCL7 (low dose = 5 ng and high dose = 20 ng, respectively) delivered on day 7 after IL-1β-BMSCs treatment significantly decreased the withdrawal threshold and withdrawal latency in a dose dependent manner (Fig. 5B and C). We next examined the effects of CCL7 on the late antinociceptive effects of IL-1β-BMSCs injected on day 7 after SNL. CCL7 levels in the dorsal horn were detected by ELISA on day 3 after IL-1β-BMSCs injection (day 10 post-SNL), when the strongest analgesic potential was achieved (refer to Fig. 2C and D). As shown in Fig. 5D, IL-1β-BMSCs treatment dramatically decreased CCL7 levels (Fig. 5D). High doses of CCL7 decreased the withdrawal threshold and withdrawal latency; however, low doses of CCL7 decreased the withdrawal threshold only (Fig. 5E and F). Although IL-1β-BMSCs injection significantly reduced SNL-induced microglia activation (Fig. 3), we did not know whether CCL7 was involved in this process. As shown in Fig. 5G and H, CCL7 significantly activated microglia that were inhibited by previous IL-1β-BMSCs treatment (Fig. 5G and H). This result indicate that the inhibitory effects of IL-1β-BMSCs treatment on microglia activation and neuropathic pain are mediated by decreasing CCL7 in the spinal cord.

### IL-1β-BMSCs inhibited astrocyte activation and reduced CCL7 level through release of IL-10 both in *vitro* and *vivo*

**Figure 6:**
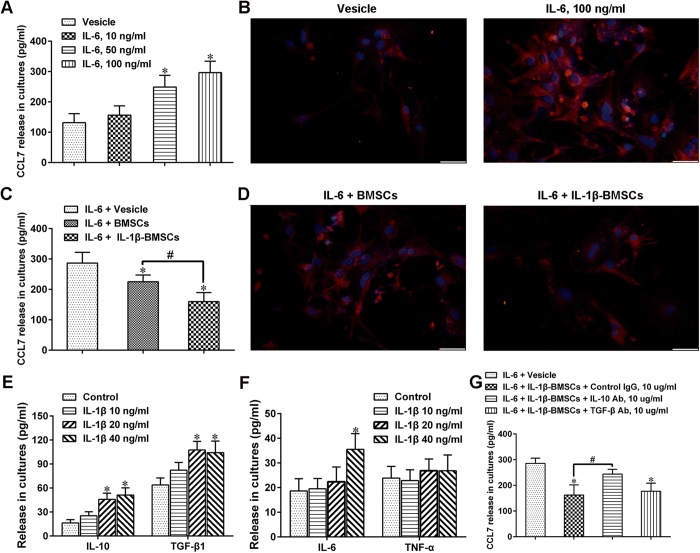
IL-1β-BMSCs inhibited CCL7 release from astrocyte through release of IL-10 *in vitro*. (**A**) CCL7 levels in the culture medium of astrocyte stimulated by various concentrations of IL-6. (**B**) Representative images of astrocyte stained by GFAP after stimulation by IL-6. Scale bars = 50 μm. (**C**) CCL7 levels in the culture medium after co-culture of IL-6 stimulated astrocytes and BMSCs or IL-1β-BMSCs. (**D**) Representative images of astrocytes stained by GFAP after co-cultured with BMSCs or IL-1β-BMSCs. Scale bars = 50 μm. (**E** and **F**) Anti-inflammatory cytokines (**E**) and pro-inflammatory cytokines (**F**) levels in culture medium of BMSCs after stimulated with various concentration of IL-1β. (**G**) IL-10 Ab, but not TGF-β Ab, reversed CCL7 inhibition of IL-1β-BMSCs *in vitro*. *P < 0.05 compared with control group or vesicle group; ^#^P < 0.05; n = 6 cultures/group. Statistical significance was determined by one-way ANOVA followed by Bonferroni’s post-hoc test. All data were expressed as the mean ± SEM.

**Figure 7:**
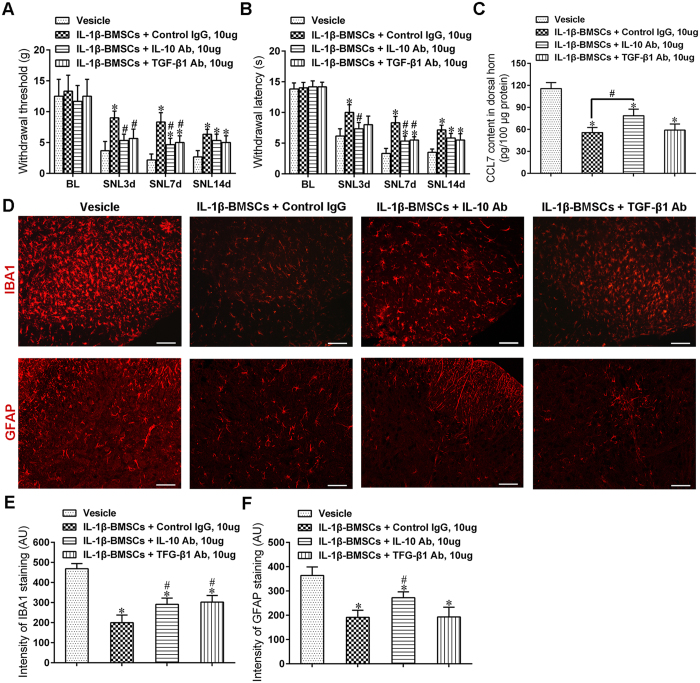
(**A** and **B**) Both IL-10 Ab and TGF-β1 Ab reversed the analgesic effect of IL-1β-BMSCs, as indicated by increased mechanical allodynia (**A**) and thermal hyperalgesia (**B**). (**C**) CCL7 levels in spinal cord after injection of IL-1β-BMSCs with IL-10 Ab or TGF-β1 Ab. (**D**) Representative immunofluorescent images of microglia (top panels) and astrocytes (bottom panels) in spinal cord after injection of IL-1β-BMSCs with IL-10 Ab or TGF-β1 Ab. Scale bars = 100 μm. (**E** and **F**) Statistic results of fluorescence intensity of IBA1 (**E**) and GFAP (**F**) in (**D**). *P < 0.05 compared with control group or vesicle group; ^#^P < 0.05; 72 rats in total and n = 6 rats/group. Statistical significance was determined by one-way ANOVA (**C**,**E** and **F**) or two-way repeated-measures ANOVA (**A** and **B**) followed by Bonferroni’s post-hoc test. All data were expressed as the mean ± SEM.

A study showed that partial sciatic nerve ligation significantly increased the expression of CCL7 in spinal astrocytes, which facilitated interaction between astrocytes and microglia, in the development of neuropathic pain^24^. To study whether the decrease in CCL7 levels in spinal cord were due to the inhibitory action of IL-1β-BMSCs on astrocytes, we activated primary astrocyte cells with IL-6, as IL-6 has been reported to mediate astrocytic release of CCL7 both *in vivo* and *in vitro*^24^. Results indicated that CCL7 levels increased significantly in a concentration dependent manner after stimulation with IL-6 (Fig. 6A). Furthermore, IL-6 stimulation also increased the expression of glial fibrillary acidic protein (GFAP) in astrocytes (Fig. 6B). In order to determine specific actions of IL-1β-BMSCs on astrocytes, we co-cultured the astrocytes with BMSCs or IL-1β-BMSCs. Results indicated that IL-1β-BMSCs had a stronger inhibitory effect on CCL7 release (Fig. 6C) and astrocyte activation (Fig. 6D) compared to BMSCs alone. In addition the levels of anti-inflammatory cytokines (IL-10 and TGF-β1) and pro-inflammatory cytokines (IL-6 and TNF-α) in supernatant were tested, with results showing that IL-1β treatment increased levels of IL-10 and TGF-β1 in a concentration dependent manner (Fig. 6E); however, IL-1β stimulation in 40 ng/ml significantly increased IL-6 levels but not TNF-α (Fig. 6F), which was consistent with the behavioral result (Fig. 1C). To further study whether IL-10 or TGF-β1 mediated the effects IL-1β-BMSCs inhibition of CCL7, we used a blocking antibody (Ab) for either IL-10 or TGF-β1 which was added into the astrocyte/BMSC’s co-culture system. We found that IL-10 Ab, but not TGF-β1 Ab significantly alleviated the inhibitory effect of IL-1β-BMSCs mediated down-regulation of CCL7 (Fig. 6G). To study whether IL-10 had similar action *in vivo*, IL-10 Ab or TGF-β1Ab was i.t. injected at a concentration of 10 μg per rat once a day from day 0 to day 6 after SNL. We found that although both IL-10 Ab and TGF-β1Ab could significantly weakened the analgesic effect (Fig. 7A and B) and microglia inhibitory effect (Fig. 7D and E) of IL-1β-BMSCs after SNL, only IL-10 Ab could reverse the inhibitory effect of IL-1β-BMSCs on astrocyte activation (Fig. 7D and F) and CCL7 levels (Fig. 7C) in spinal cord. Taken together, these results indicate that IL-10 is involved in the inhibitory effect of IL-1β-BMSCs mediated astrocyte activation and CCL7 release.

### Directional migration of IL-1β-BMSCs to the ipsilateral spinal cord is mediated at least partly through CXCL13

**Figure 8:**
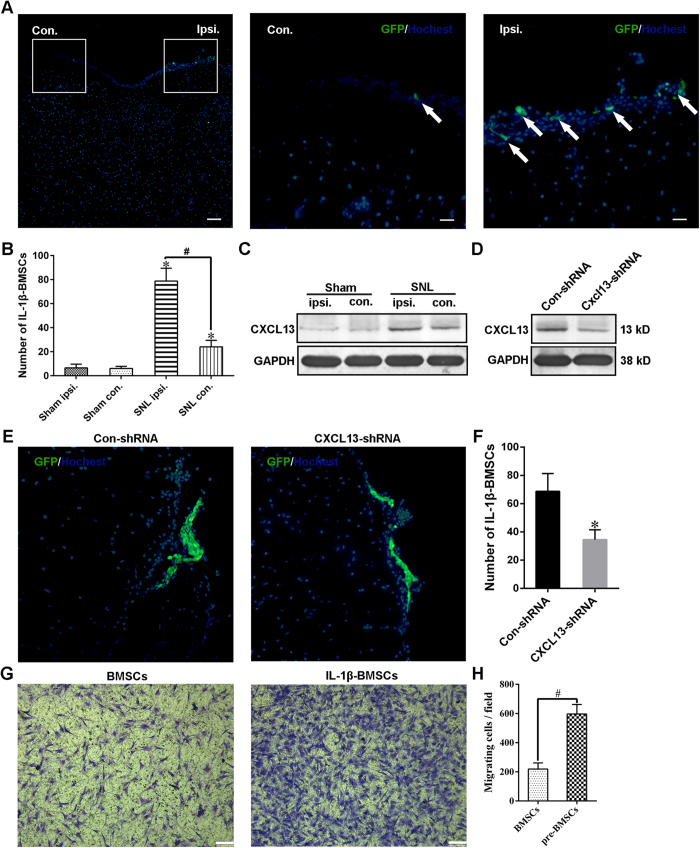
CXCL13 mediated the directional migration of IL-1β-BMSCs *in vivo* and *in vitro*. (**A**) Representative immunofluorescent images of the spinal cord on day 7 after SNL (day 7 after IL-1β-BMSCs injection). The middle panel and the right panel were the enlarged images of the white box parts in the left panel. Arrows in the middle and right panels are GFP-labeled IL-1β-BMSCs (GFP-BMSCs). Scale bars = 200 μm (left panel) and 50 μm (middle and right panels). (**B**) Numbers of GFP-BMSCs in ipsilateral and contralateral spinal cords on day 7 after SNL or sham operation. (**C**) Expression of CXCL13 in ipsilateral and contralateral spinal cords seven days after SNL or sham treatment. (**D**) Expression of CXCL13 in ipsilateral spinal cords on day 7 after SNL. Control or Cxcl13 shRNA lentivirus vectors were injected three days before SNL. Full-length bolts in C and D were presented in Supplementary Figure S1. (**E**) Representative immunofluorescent images of GFP-BMSCs on the surface of an ipsilateral spinal cord. (**F**) Statistical results of the numbers GFP-BMSCs on the surface of ipsilateral spinal cords. (**G**) Representative images of transwell chemotaxis of BMSCs and IL-1β-BMSCs toward CXCL13. Scale bars = 100 μm. (**H**) Statistical results of cell migration. For (**A**–**F**), 32 rats in total and n = 7–8 rats/group (4 rats for immunofluorescence and 3–4 rats for western blot), and for (**H**) ten random fields at 100 × magnification were analyzed, n = 5 cultures/group. *P < 0.05 compared with the sham group or con-shRNA group, ^#^P < 0.05. Statistical significance was determined using one-way ANOVA followed by Bonferroni’s post-hoc test for (**B**) or a Student’s t test for (**F** and **H**). All data were expressed as the mean ± SEM.

We found that GFP labeled IL-1β-BMSCs (GFP-BMSCs) directionally migrated to the ipsilateral spinal cord after SNL (Fig. 8A and B); however, the mechanism for this is unknown. Although a previous study showed that the CXCL12/CXCR4 axis regulated the migration of BMSCs to lumbar DRGs after CCI in mice^5^, the action of other chemotactic factors to the migration of BMSCs remains unknown. A recent study indicated CXCL13 was significantly increased in the spinal cord neurons after SNL, and played a key role in the development of neuropathic pain^8^. In addition, as CXCL13 also played a major role in the osteogenic differentiation of BMSCs^33^, we tested whether CXCL13 mediated the directional migration of injected IL-1β-BMSCs. CXCL13 was significantly upregulated in the ipsilateral spinal cord after SNL (Fig. 8C), and i.t. injection of IL-1β-BMSCs did not affect the levels of CXCL13 in spinal cord (Supplementary Figure S1C). Moreover, following injection of Cxcl13 shRNA lentivirus vectors (Cxcl13 shRNA, Supplemental Table S2), and subsequent knockdown of CXCL13, there were significantly reduced the numbers of GFP-BMSCs on the surface of ipsilateral spinal cord (Fig. 8D,E and F). Our cell experiments also showed that IL-β pre-treatment significantly increased the numbers of BMSCs migrating toward CXCL13 stimulation (Fig. 8G and H). Additionaly, IL-1β pre-treatment did not upregulate the expression of CXCR5 or CXCL13, but it focused CXCR5 expression within the periphery of the nucleus (Supplementary Figure S1A and B). Taken together these results indicate that directional migration of IL-1β-BMSCs is mediated by increased CXCL13 expression in spinal cord after SNL. However, the specific mechanism needed further studies.

## Discussion

Neuropathic pain is closely associated with neuroinflammation, which is mediated by neutrophils, macrophages, microglia, mast cells and lymphocytes^34^. Many studies have shown that BMSCs are able to directionally migrate to sites of inflammation and inhibit the inflammatory response and significantly alleviate neuronal damage in many nervous system diseases, such as spinal cord lesion^17^,^20^. Additionally, a growing body of evidences indicate that system wide or local injection of BMSCs ameliorates neuropathic pain. However, whether IL-1β pre-treated BMSCs have a better analgesic effect is still unclear. In the current study, we found that BMSCs pre-treated with different concentrations of IL-1β remarkably increased the withdrawal threshold and withdrawal latency, indicating amelioration of mechanical allodynia and thermal hyperalgesia, respectively. Moreover, we concluded that BMSCs pre-treated with 20 ng/ml of IL-1β had the strongest analgesic effect.

Mechanical allodynia and heat hyperalgesia are the two major characteristics of neuropathic pain with the early phase considered to be three days post injury and the late phase considered to be seven days post injury^35^. Our findings showed that i.t. injection of BMSCs or IL-1β-BMSCs in rats ameliorated mechanical allodynia and hyperalgesia, regardless of phase stage. This result was consistent with the study by Gang Chen *et al*.^5^, in which the authors showed that i.t. injection of BMSCs relieved neuropathic pain both in the early and late phases for more than five weeks after CCI in mice. Moreover, IL-1β-BMSCs further increased the withdrawal threshold and withdrawal latency, which indicated a stronger relief of neuropathic pain. However, the effectiveness of early IL-1β-BMSCs injection was more prominent than BMSCs alone for at least seven days, while the effectiveness of late IL-1β-BMSCs injection was more prominent for only five days, thus implying that IL-1β-BMSCs played a greater inhibition on the initiation, rather than the maintenance of neuropathic pain.

It remains unclear whether the IL-1β-BMSCs had greater inhibition on neuroinflammation compared to BMSCs alone. As microglia play a major role in the initiation and maintenance of neuropathic pain^36^,^37^,^38^, we detected the effects of IL-1β-BMSCs on microglia. Results indicated that both early or late injection of IL-1β-BMSCs decreased the number of microglia in the spinal cord after SNL compared to BMSCs alone. As IL-1β and TNF-α are the most common proinflammatory cytokines in many diseases^30^,^39^, we chose to evaluate the action of IL-1β-BMSCs on the levels of these two cytokines in spinal cord. We found that both BMSCs and IL-1β-BMSCs decreased the levels of IL-1β and TNF-α; however, i.t. injection of IL-1β-BMSCs had a stronger inhibition on IL-1β but not TNF-α compared to BMSCs alone. These results indicate that the more prominent analgesic effects of IL-1β-BMSCs may be related with the down regulation of IL-1β. Interestingly, a recent study showed that IL-18 was closely related with the development of neuropathic pain^22^,^31^,^32^, and our results indicated that IL-1β-BMSCs significantly reduced the level of IL-18 in the spinal cord after SNL. As IL-1β and IL-18 are known to be secreted by microglia^22^,^31^, we hypothesize that the greater anti-inflammatory effects of IL-1β-BMSCs may be attributed to inhibition of microglial activation.

Although the exact mechanisms by which IL-1β-BMSCs inhibit microglia activation remain unknown, many studies have shown that several chemokines play an important role in neuronal-glial interactions and contribute to the development of neuropathic pain^8^,^13^,^40^. CCL2 and CCL7 are the two most common activators of microglia in the development of neuropathic pain^24^,^25^. CCL7 is primarily expressed in astrocytes after partial sciatic nerve injury, and can activate microglia through the CCR2 receptor^24^. Our current study indicated that CCL7, but not CCL2, mediated the analgesic effects of IL-1β-BMSCs. Firstly, i.t. injection of IL-1β-BMSCs significantly decreased the levels of CCL7 rather than CCL2 in the spinal cord after SNL and secondly, CCL7 injection significantly reversed the analgesic effects of IL-1β-BMSCs. Moreover, CCL7 also reversed the microglial inhibition of IL-1β-BMSCs, thus implying that IL-1β-BMSCs suppressed the activation of microglia, at least partly through a reduction in CCL7 levels.

IL-6 is known to promote astrocyte derived CCL7 release^24^. Our results showed that IL-6 could activate astrocytes and promote CCL7 release in a concentration dependent manner. IL-1β-BMSCs were found to have a stronger inhibitory effect on astrocyte activation and CCL7 release compared to BMSCs alone *in vitro*. In addition, IL-1β pre-treated BMSCs secreted more IL-10 and TGF-β1 than the BMSCs alone, with results indicating that IL-10, rather than TGF-β1, inhibited astrocyte activation and CCL7 release *in vitro*. Through i.t. injection of IL-10 Ab or TGF-β1 Ab, results indicated that both IL-10 Ab and TGF-β1 Ab could revised the inhibitory effect of IL-1β-BMSCs on neuropathic pain and microglia activation, but only IL-10 Ab could reviesed their inhibitory effect on astrocyte activation and CCL7 levels in spinal cord. CCL7 can activate microglia through CCR2 and promote neuropathic pain after partial sciatic nerve ligation^24^, our results showed that i.t. injection of exogenous CCL7 could reverse the inhibitory effect of IL-1β-BMSCs on pain and microglia activation. Previous study showed that i.t. injection of BMSCs inhibited neuropathic pain via TGF-β1^5^; however, our results indicated that IL-1β-BMSCs could inhibit microglia activation and neuropathic pain not only by releasing TGF-β1, but also by decreasing CCL7 level in spinal cord, which may be mediated through release of IL-10 and inhibition of astrocytes. Our study provides a possible explanation why IL-1β pre-treatment can enhance the inhibitory effect of BMSCs on microglia activation and neuropathic pain.

In addition, we found that IL-1β-BMSCs directionally migrated to the surface of the ipsilateral spinal cord after SNL. Although a study showed that the CXCL12/CXCR4 axis mediated the migration of BMSCs to lumbar DRG after CCI in mice^5^, the mechanism controlling IL-1β-BMSCs migration remains unclear. A recent study indicated that the levels of neuronal derived CXCL13, a chemokine known to play a key role in the development of neuropathic pain, greatly increased in the spinal cord after peripheral nerve injury^8^. Moreover, CXCL13 is known to promote osteogenic differentiation of BMSCs^33^, and therefore we set out to determine whether CXCL13 was involved in the directional migration of IL-1β-BMSCs. We found that expression of CXCL13 significantly increased on the ipsilateral spinal cord after SNL, and knockdown of CXCL13 using shRNA lentivirus vector significantly decreased the number of IL-1β-BMSCs on the surface of the ipsilateral side of the spinal cord. Moreover, we also found that IL-1β pre-treatment significantly improved the migration ability of BMSCs toward the direction of CXCL13 *in vitro*. IL-1β treatment neither increased the expression of CXCR5 nor increased the levels of CXCL13 from BMSCs, but instead focused CXCR5 expression to the periphery of the BMSCs nucleus, which further proved the action of CXCL13 in migration of IL-β-BMSCs. In addition, the CXCL 13 levels in the spinal cord significantly increased after SNL, and i.t. injection of IL-1β-BMSCs did not affect CXCL13 level after SNL. Taken together, directional migration of IL-1β-BMSCs was mediated by CXCL13 increases in the spinal cord after SNL. Further studies are needed in order to elucidate the specific mechanism involved in this directional migration.

In conclusion, our study showed that i.t. injection of IL-1β-BMSCs could inhibit microglia activation and neuropathic pain not only by releasing TGF-β1, but also by decreasing CCL7 levels in the spinal cord after SNL, which maybe mediate by release of IL-10 and inhibition of astrocytes. In addition, directional migration of IL-1β-BMSCs was mediated by CXCL13, increasing in spinal cord after SNL. Further studies are needed to explore the specific mechanisms involved in each of these findings.

## Methods

### Experimental Animals

All animal experiments were reviewed and approved by the Institutional Animal Care and Use Committee of the Second Military Medical University and followed the guidelines for the study of pain in awake animals established by the International Association for the Study of Pain^41^. Male Sprague Dawley rats (weight 200 ± 20 g) were provided by the Animal Experiments Center of the Second Military Medical University (Shanghai, China) were used for all subsequent experiments.

### Isolation, culture and characterization of BMSCs from GFP Sprague Dawley rats

Eight-week-old male SD rats that over express GFP were anaesthetized with 10% chloral hydrate and primary BMSCs were isolated and cultured as previously described^19^. Briefly, the bone marrow in long bones (femur and tibia) was dissected, homogenized and flushed by 0.01 M phosphate buffered saline (PBS) using a 20 ml syringe, then the collected bone marrow was filtered (70 μm) in order to remove fragments. The harvested cells were isolated by Ficoll density gradient centrifugation (1.077 g/ml; Sigma-Aldrich, Steinheim, Germany) at 400 g for 20 minutes. Cells in the interface were collected and washed with Dulbecco’s Modified Eagle Medium/Nutrient Mixture F-12 (DMEM/F-12, Gibco, Grand Island, NY, USA), then centrifuged at 600 g for 10 minutes. The harvest cells were resuspended in complete medium (DMEM/F-12 with 10% foetal bovine serum [FBS, Gibco], 100 μg/ml streptomycin and 100 U/ml penicillin). Cells were plated in T75 cell culture flask (NEST, Wuxi, China), and then incubated in a humidified atmosphere at 37 °C with 5% CO_2_. After 4–5 days, medium was changed and non-adherent cells were removed. The medium was changed once every 3–4 days. When 80% of the flask was covered, BMSCs were digested, centrifuged, and replaced at a density of 5000 cells/cm^2^. Cells at the 3rd passage were tested by flow cytometric analysis to examine the expression of the surface markers, CD29, CD90, CD31 and CD45 (BD Biosciences, San Jose, CA, USA). The phenotypic characterization of BMSCs was confirmed by the presence of CD29 and CD90, and the absence confirmed by the expression of pan-hematopoietic markers CD31 and CD45. The osteogenic and adipogenic differentiation potential of the cells were also examined by Alizarin red staining and Oil Red staining. Finally, cells were stained for vimentin (V9) (Abcam, Cambridge Science Park, UK). For more information, please refer to the supplementary methods section.

### Pre-stimulation of BMSCs

BMSCs at the 4^th^ passage were divided into four groups and stimulated with IL-1β (Sigma, St. Louis, MO, USA) in serum free DMEM/F12 (medium only) at concentrations of 10, 20, and 40 ng/ml, respectively, for 24 h, after which time the medium was removed and replaced with complete DMEM/F12 and cells were cultured for a further 24 h.

### Astrocyte cultures and treatment

As described previously, astrocytes were acquired from neonatal rats (P2–3)^42^. The mixed cells were plated onto culture flasks which were coated with poly-L-lysine, and cultured in DMEM/F12 (Gibco) containing 10% FBS (Gibco). The medium was changed every 2–3 days, and 6–10 days later, the cultures were put on shaker (2.5 g, 37 °C, 5% CO_2_) overnight to remove microglia and oligodendrocyte lineage cells. The astrocytes were harvested and plated (1 × 10^5^/cm^2^) onto coverslips in 12-well plates for further analysis. Cells were 24 h before treatment. For IL-6 studies, astrocytes were treated with IL-6 (Sigma) at concentrations of 0, 10, 50, 100 ng/ml for further 24 h, and the level of GFAP and CCL7 were analyzed to acquire the optimum concentration of IL-6. In further studies, the optimum concentration of IL-6 was chosen to stimulate astrocytes.

Co-cultures of pure astrocytes (1.0 × 10^5^/cm^2^) and BMSCs or IL-1β-BMSCs (1.0 × 10^5^/cm^2^) were plated onto the coverslips in 24-well plates for 24 h after which IL-6 (100 ng/ml) was added. Cells were cultured for a further 24 h and the levels of GFAP and CCL7 were analyzed. For Ab blocking studies, IL-10 blocking Ab (BioLegend, San Diego, USA) or TGF-β1 neutralizing antibody (R&D Systems, Minneapolis, MN, USA) were added to the co-culture system 0.5 h before IL-6 stimulation.

### Preparation of the pain model and intrathecal catheter

Rats (180–220 g) were choen for SNL operation if their mechanical paw withdrawal thresholds were between 8 and 15 g. The SNL neuropathic pain model was performed as previously described^29^. Briefly, selected rats were anesthetized with 10% chloral hydrate (0.3 ml/100 g) and after preoperative skin preparation, rats were put in the prone position. An incissation was made to expose the spine, the left transverse process was removed, and the L5 nerve was isolated and tightly ligated with black silk. The muscle and skin were then sutured for the completion of the operation. Implantation of intrathecal cannula was performed following methods previously described^43^. A polythene PE10 intrathecal catheter (PORTEX, Smiths Medical, NH, USA; approximately 16 cm in length, with a volume of 10 μl and an inner diameter of 0.28 mm and an outer diameter of 0.61 mm) was placed under the skin from lower back to the head, forming a channel linking the cisterna terminalis to exterior for i.t. injection. Rats were omitted from subsequent experiments if limb dysfuction or infection of the incissation area was evident following SNL. Additionally, rats were omitted if there were complications inserting the intrathecal cather. For more information regarding the intrathecal catheter, please refer to the supplementary methods section. For quality control, all operations were performed by the same experimental team with the exact same methods.

### Cell transplantation and drug administration

Experimental animals were divided into four groups: negative control (sham-operated group), positive control (SNL operation group injected with phosphate-buffered saline (PBS)), BMSCs group (SNL operation group injected with BMSCs) and IL-1β-BMSCs group (SNL operation group injected with stimulated BMSCs). Each group consisting of eight rats (in an operational situation, six more rats received SNL in case of accidental death or failure of operation).

Before transplantation, GFP labeled BMSCs were divided into two groups: one group stimulated with 20 ng/ml of IL-1β for 24 h and the other without IL-1β stimulation. After 24 h, cells from both groups were trypsinized (0.25%) without ethylene diamine tetraacetic acid (EDTA, Gibco) for 2–3 min. After neutralization by complete medium, cells were diluted with 0.01 M PBS to a final concentration of 10^8^ cells/ml. Stimulated or un-stimulated BMSCs or 0.01 M PBS (negative control) at a volume of 25 μl (about 2.5 × 10^6^ cells) were i.t. injected into rats through thePE10 catheter, after which an additional 10–15 μl of PBS was injected in order to move cells in the catheter into the lumbar cistern. For Ab blocking studies, IL-10 blocking Ab or TGF-β1 blocking Ab was given once a day from day 0 to day 6 after SNL.

### Lentiviral vectors production

Three shRNAs targeting rat Cxcl13 and one control shRNA were designed, and the recombinant lentivirus containing Cxcl13 shRNA or control shRNAs was packaged using pGCSIL-GFP vector by Shanghai GeneChem and the sequences were shown in Supplemental Table S2.

### Pain threshold assessment

Mechanical paw withdrawal thresholds were evaluated by measuring the withdrawal response after stimulation of the left sole (operational sides) with Von Frey filaments (Stoelting, Wood Dale, IL, USA)^29^. Pressure was exerted until the Von Frey filament was slightly bent and the stimulation was gradually increased. A positive response was classified as dodging, shaking, or flinching from the stimulation and all stimulation responses were recorded. The measurements were repeated five times and performed between 11 a.m. and 3 p.m. The pain threshold was recorded in grams (g).

Thermal hyperalgesia was tested as previously described^5^. A hargreaves radiant heat apparatus (IITC Life Science, Woodland Hills, CA, USA) was used, and the radiant heat source was adjusted to produce a baseline latency between 11–14 s. A cutoff point was set at 20 s to avoid tissue damage. Rat motor function was tested using a rotarod system (IITC Life Science).

The SNL model is a classic neuropathic pain model where by the pain threshold decreases from the first day after SNL, and is maintained for several weeks^8^,^9^,^10^. In order to determine the most suitable concentration of IL-1β, we preliminarily chose the time point of 7 days and 14 days after SNL (IL-1β-BMSCs i.t. injection) to estimate the effect of IL-1β-BMSCs on pain alleviation (Fig. 1C). IL-1β-BMSCs were i.t. injected just before SNL or on day 7 after SNL in order to evaluate their effect on pain prevention (Fig. 2A and B) or pain treatment (Fig. 2C and D), and the time points of 1d, 3d, 5d, 7d, 10d, 14d after i.t. injection were chosen. According to Fig. 2, IL-1β-BMSCs i.t. injected prior to SNL had the strongest analgesic effect on day 7 after injection (SNL 7d) and on day 3 after injection (SNL 10d) for the group receiving i.t. injection on day 7 after SNL. These two time points (SNL 7 d or SNL 10 d) were thus chosen for i.t. injection of CCL7 (Fig. 5).

### Tissue collection

For behavioral analysis (Fig. 2A–D) for rats i.t. injected prior to SNL, tissue was collected on day 7 after injection (Fig. 3A and B, Fig. 4, Fig. 5A, Fig. 6L and Fig. 7A–F), and for rats i.t. injected on day 7 after SNL, tissue was collected on day 3 after injection (Fig. 3C and D, Fig. 5D).

For western blot analysis, rats were anaesthetized with 10% chloral hydrate and the lumbar enlargements were carefully isolated. Both ipsilateral and contralateral spinal cords were collected and stored in microtubes at −80 °C until further use.

For immunofluorescence, rats were anaesthetized with 10% chloral hydrate and then with 0.01 M PBS followed by 4% paraformaldehyde (dissolved in PBS). Immediately after perfusion, the spinal cord was isolated and post-fixed overnight in 4% paraformaldehyde at 4 °C. Tissue was dehydrated in 10% sucrose solution and 20% sucrose solution, consecutively, and the lumbar region of each spinal cord (L4-L6) was quickly frozen (3 min in isopentane at −80 °C) and stored at −80 °C until cryosectioning. Three to four slices/rat were choen for immunofluorescence.

### Western blot

The lumbar segment of ipsilateral spinal cord was homogenized in lysis buffer (Beyotime Biotechnology, Shanghai, China) containing protease inhibitors phenylmethanesulfonyl fluoride (PMSF). Triton X-100 was added, and the homogenates were incubated on ice for 30 min followed by centrifugation at 20,000 × *g* for 15 min. Protein concentrations were quantified and run through a 12% polyacrylamide gel and transferred to a 0.4 μm polyvinylidene fluoride membrane. Membranes were then blocked with milk and incubated with rabbit anti-CXCL13 (1:1000, Abcam) for 16 h at 4 °C. Primary antibody was removed a goat anti-rabbit secondary antibody conjugated with horseradish peroxidase (HRP, 1:1000, Sigma) was added for 1 h at room temperature away from any light. Target protein was examined by an enhanced chemiluminescence detection system (Pierce, USA) according to the manufacturer’s instructions. Samples were normalized with a monoclonal GAPDH antibody produced in mouse (1:1000, Abcam). Image J software (NIH, USA) was used to detect the signal intensity. For more information regarding the western blot, please refer to the supplementary methods section.

### Immunofluorescence

#### GFP BMSCs detection

As injected BMSCs could not pass through the arachnoid of the spinal cord, most cells were on the surface of arachnoid, and therefore samples were immediately frozen after perfusion, processed for cryosectioning as above and stained with 4′,6-diamidino-2-phenylindole (DAPI, Sigma-Aldrich). For the *in vitro* study, untreated BMSCs and IL-1β-BMSCs were stained with primary antibodies against CXCL13 (rabbit anti- rat CXCL13, 1: 400, Abcam) and CXCR5 (rabbit anti- rat CXCR5, 1: 400, Abcam) for 16 h at 4 °C, and then incubated with secondary antibody (goat anti-rabbit-Dylight488 antibody, 1:500, Invitrogen, Carlsbad, CA, USA) for 1 h at room temperature.

#### Microglia and astrocyte detection

Frozen samples for immunofluorescence were serially cut at −30 °C to obtain ten series of 20 μm thick transverse sections, which were collected on Thermo Scientific super frost plus glass slides (VWR International, Leuven, Belgium). For cell samples, cells were fixed with 4% paraformaldehyde in PBS for 20 minutes, and then washed 0.01 M PBS for three times. IBa1 or GFAP staining was performed as previously descrived^44^. Briefly, sections were incubated with a primary antibody against IBA1(rabbit anti-rat Iba1, 1:1,000, Abcam) or GFAP (mouse anti-rat GFAP, 1:500, Abcam) for 16 h at 4 °C, and then incubated with secondary antibodies (goat anti-rabbit-Dylight488 antibody, 1:500, Invitrogen; goat anti-mouse-Dylight488 antibody, 1:500, Invitrogen) for 1 h at room temperature. All digitized images displaying immunopositivity were obtained using a fluorescence microscope (Olympus AX80; Olympus Optical, Tokyo, Japan). For more information regarding the microglia and astrocyte detection, please refer to the supplementary methods section.

#### qRT-PCR

Total RNA was isolated from spinal tissue or adherent cells using TRIzol reagent (Invitrogen) and RNA concentration was measured photometrically. cDNA synthesis was carried out using RevertAid First Strand cDNA Synthesis Kit (Fermentas, Ontario, Canada) according to the manufacturer’s instructions. Analysis was performed using qRT-PCR with gene-specific primers (see Supplementary Table S1) on a MyiQ™ (Bio-Rad, Hercules, CA, USA) with SYBR Green Real-time PCR Master Mix (TOYOBO Biotech, Osaka, Japan). Amplification of target cDNA was normalized to *GAPDH* expression. Relative levels of target mRNA expression were calculated using the 2−ΔΔCt method.

#### ELISA kit

Tissue samples were suspended in 200 μl lysis buffer (Cell Signaling, Germany) and 2 μl PMSF (200 mM), according to the manufacturer’s instructions, incubated on ice for 5 min, and sonicated and cleared by centrifugation (14,000 × *g* for 10 min at 4 °C). Supernatants were collected and protein content was determined using a MicroBCA assay (Thermo Scientific, Germany). IL-1β, IL-18, TNF-α and CCL7 were measured using respective ELISA systems (R&D Systems, Wiesbaden, Germany) according to the manufacturer’s instructions.

#### Transwell plates

BMSCs were divided into two groups: IL-β-BMSCs group (BMSCs pre-treated with 20 ng/ml of IL-β for 24 h) and the ordinary BMSCs group (BMSCs alone). All cells were digested with 0.25% trypsin without EDTA (Gibco) for 2–3 min and after neutralization by complete medium, IL-β-BMSCs and ordinary BMSCs were cultured in transwell plates at a density of 10^4^ cells/well. Cell were then stimulated with CXCL13 (200 ng/ml) for 24 h and migrating BMSCs were detected with crystal violet staining. The numbers of cells were counted on membranes that were transferred on glass slides and mounted with mounting medium. Five replicate wells were used for each group, and two–three fields were taken at 100× magnification for each well.

### Statistical Analysis

Differences between groups were examined for statistical significance using one‐way factorial analysis of variance (ANOVA) or two-way repeated-measures ANOVA or the Student’s t test. P < 0.05 was taken to be statistically significant, for comparisons between more than two groups, * and # were used to describe the differences, respectively. The above tests were conducted using SPSS software version 21.0 (SPSS, Chicago, IL, USA). GraphPad Prism software, and version 5.00 (GraphPad software, San Diego, CA, USA) was used for drawings.

## Additional Information

**How to cite this article:** Li, J. *et al*. Interleukin-1β pre-treated bone marrow stromal cells alleviate neuropathic pain through CCL7-mediated inhibition of microglial activation in the spinal cord. *Sci. Rep.* **7**, 42260; doi: 10.1038/srep42260 (2017).

**Publisher's note:** Springer Nature remains neutral with regard to jurisdictional claims in published maps and institutional affiliations.

## Supplementary Material

Supplementary Information
