# Supplementary material for: Interleukin-1β pre-treated bone marrow stromal cells alleviate neuropathic pain through CCL7-mediated inhibition of microglial activation in the spinal cord
Source: Sci Rep. 2017 Feb 14;7:42260. doi: 10.1038/srep42260 (PMC5307320; doi:10.1038/srep42260)
Supplement: Supplementary Information [file srep42260-s1.pdf]

# Interleukin-1β pre-treated bone marrow stromal cells alleviate neuropathic pain through CCL7-mediated inhibition of microglial activation in the spinal cord

Jian Li[1a], Guoying Deng[2a], Haowei Wang[1a], Mei Yang[1], Rui Yang[1], Xiangnan Li[3], Xiaoping Zhang[4*], Hongbin Yuan[1*]

[1]Department of Anesthesiology, Changzheng Hospital, Second Military Medical University, 415 Fengyang Road, Shanghai, 200003, China

[2]Trauma Center, Shanghai General Hospital, Shanghai Jiaotong University School of Medicine, 650 Xin Songjiang Road, Shanghai, 201620, China.

[3]Department of Anesthesiology, the Third People's Hospital of Yancheng, Yancheng, 224001, China

[4]Department of Interventional & Vascular Surgery, Tongji University School of Medicine, Shanghai 200072, China

[a]These authors contributed equally to this work and should be considered co-first authors

[*]Corresponding Authors:

Hongbin Yuan

Department of Anesthesiology, Changzheng Hospital, Second Military Medical University, 415 Fengyang Road, Shanghai, 200003, China. E-mail: jfjczyy@aliyun.com.Tel: (8621)81885822; Fax: (8621)81885822

Xiaoping Zhang

Department of Interventional & Vascular Surgery, Tongji University School of Medicine, Shanghai 200072, China. E-mail: zxpsibs@163.com. Tel: 86-21-64373365; Fax: 86-21-64373365

## Supplementary methods

**Isolation, culture and characterization of BMSCs from GFP Sprague Dawley rats**.

Eight-week-old male SD rats that over express GFP were anaesthetized with 10% chloral hydrate and primary BMSCs were isolated and cultured as previously described. Briefly, the bone marrow in long bones (femur and tibia) was dissected, homogenized and flushed by 0.01M PBS using a 20 ml syringe, then the collected bone marrow was filtered (70μm) in order to remove fragments. The harvested cells were isolated by Ficoll density gradient centrifugation (1.077 g/ml; Sigma-Aldrich, Steinheim, Germany) at 400 g for 20 minutes. Cells in the interface were collected and washed with Dulbecco's Modified Eagle Medium/Nutrient Mixture F-12 (DMEM/F-12, Gibco, Grand Island, NY, USA), then centrifuged at 600 g for 10 minutes. The harvest cells were resuspended in complete medium (DMEM/F-12 with 10% foetal bovine serum (FBS, Gibco, USA), 100 μg/ml streptomycin and 100 U/ml penicillin). Cells were plated in T75 cell culture flask (NEST, Wuxi, China), and then incubated in a humidified atmosphere at 37℃ with 5% $CO_2$. After 4-5 days, medium was changed and non-adherent cells were removed. The medium was changed once per 3-4 days. When 80% area of flask was

covered, BMSCs were digested, centrifuged, and replaced at a density of 5000 cells/cm$^2$.

Cells at the 3rd passage were tested by flow cytometric analysis to examine the expression of the surface markers,CD29, CD90, CD31 and CD45 (BD Biosciences, San Jose, CA, USA), as the phenotypic characterisation of BMSCs were confirmed on the presence of CD29, CD90 and the absence pan-hematopoietic marker CD31, CD45. The osteogenic and adipogenic differentiation potential of the cells were also examined by Alizarin red staining and Oil Red staining.

Adipogenesis cells were seeded at 50,000 cells/well in a six-well plate in complete medium. When cells reached sub-confluence, the medium was changed to complete medium supplemented with 1 µmol/L dexamethasone, 60 µmol/L indomethacin and 50 µmol/L IBMX for 14 days. Adipocyte differentiation was quantified by the formation of lipid droplets visualized by OIL RED O stain in an inverted light microscope.

Osteogenesis cells were seeded at 50,000 cells/well in a six-well plate. When cells reached sub-confluence, medium was changed to DMEM/F12 10% FBS, supplemented with 0.1 µmol/L dexamethasone and 50 µg/mL ascorbic acid for 21 days. Osteogenesis was quantified using Alizarin Red S positive staining.

Finally, cells were stained for vimentin (V9) (Abcam, Cambridge Science Park, UK). Cells were rinsed three times with ice-cold PBS and fixed with 4% paraformaldehyde in PBS and permeated with 0.5% Triton X-100 in PBS for 10 min. Samples were then incubated with specific primary antibodies overnight at 4°C. After extensive washes, samples were stained with secondary antibody IgG conjugated with Alexa 594 nm (Invitrogen, Carlsbad, CA, USA) and Hoechst 33258 (10 µg / ml) for 1 hour at room temperature. Finally, samples were washed again in PBS, fixed and imaged using a fluorescence microscope (Olympus AX80; Olympus Optical, Tokyo, Japan).

**Implantation of intrathecal cannula**

Implantation of intrathecal cannula was performed following methods previously described. Rats were anaesthetized with sodium pentobarbital and the L4 - L5 spinal processes were exposed and the muscles beside the processes were reflected. A PE-10 polyethylene tubing was inserted into the cavum subarachnoidale through the interval between L4 and L5 vertebrate, approximately 1 cm until the tip of the tubing reached the lumbar cisterna. The outer part of the tubing was fixed onto the muscle of the lumbar and the skin of the head. A small volume of lidocaine, approximately 10 µL, was administered in the tubing followed by 10 µL artificial cerebrospinal fluid and the rat exhibited motor dysfunction, which indicated the successful implantation of the intrathecal catheter.

**Microglia and astrocyte detection**

Frozen samples for immunofluorescence were serially cut at −30°C to obtain ten series of 20 µm thick transverse sections, which were collected on Thermo Scientific superfrost plus glass slides (VWR International, Leuven, Belgium). For cell samples, cells were fixed with 4% paraformaldehyde in PBS for 20 minutes. Tissues or cellsstaining was performed as previously described. Briefly, after thawing and drying overnight at 37°C, sections were washed three times (5 min each) in PBS before incubation in a blocking solution (5% FBS, 1% Triton in PBS) for 90 min at room temperature (RT). Sections were incubated with primary antibody against IBA1(rabbit anti-rat Iba1, 1:1,000, Abcam) or GFAP (mouse anti-rat GFAP, 1:500, Abcam) diluted in PBS containing 1% HS and 1% Triton overnight at 4°C. The next day, sections were washed three times (10 min each) in PBS, and incubated with the secondary antibody (goat anti-rabbit-Dylight488 antibody, 1:500, Invitrogen) or second antibody (goat anti-mouse-Dylight488 antibody, 1:500, Invitrogen) diluted in the same working solution for 1 h at room temperature. After three washes in PBS (5 min each), nuclei were stained with DAPI (1:5,000, Sigma-Aldrich) for 15 min at room temperature and embedded in glycerol and cover-slipped.

All digitized images of immunopositivity were obtained using a fluorescence microscope (Olympus AX80; Olympus Optical). Ten randomly chosen sections from each group were used for comparison, and from each section three randomly chosen fields of 200 × magnification were counted.

**Western blot**

Animals were deeply anaesthetized using 10% chloral hydrate (0.5 ml/100 g body weight) and decapitated. The lumbar segments of rat spinal cords were dissected out immediately on ice in order to reduce the degradation of protein. The lumbar segments of ipsilateral spinal cords were homogenized in a lysis buffer (Beyotime Biotechnology, Shanghai, China) containing protease inhibitors phenylmethanesulfonyl fluoride (PMSF) and 1% Triton X-100. Homogenates were incubated on ice for 30 min followed by separation via centrifugation (20,000*g*× 15 min). Protein was quantified using the BCA Protein Assay (Pierce, Rockford, IL, USA). The supernatant was run through a 12% polyacrylamide gel, wet electro-transferred to a 0.4 μm polyvinylidene fluoride membrane and blocked in 10% evaporated skimmed milk for 1 h at room temperature on a rocker. Membranes were incubated overnight (approximately 16 h at 4°C in rabbit anti-CXCL13 (1:1000, Abcam). Blots were rinsed with 0.01 M PBS three times (10 min each) and incubated in a goat anti-rabbit secondary antibody conjugated with horseradish peroxidase (HRP, 1:1000, Sigma) for 1 h at room temperature. Target proteins were examined using an enhanced chemiluminescence detection system (Pierce) and all samples were normalized with a monoclonal GAPDH antibody produced in mouse (1:1000, Abcam). Specific bands were estimated by molecular weight sizes and Image J software (NIH, Bethesda, MD, USA) was used to detect the signal intensity.

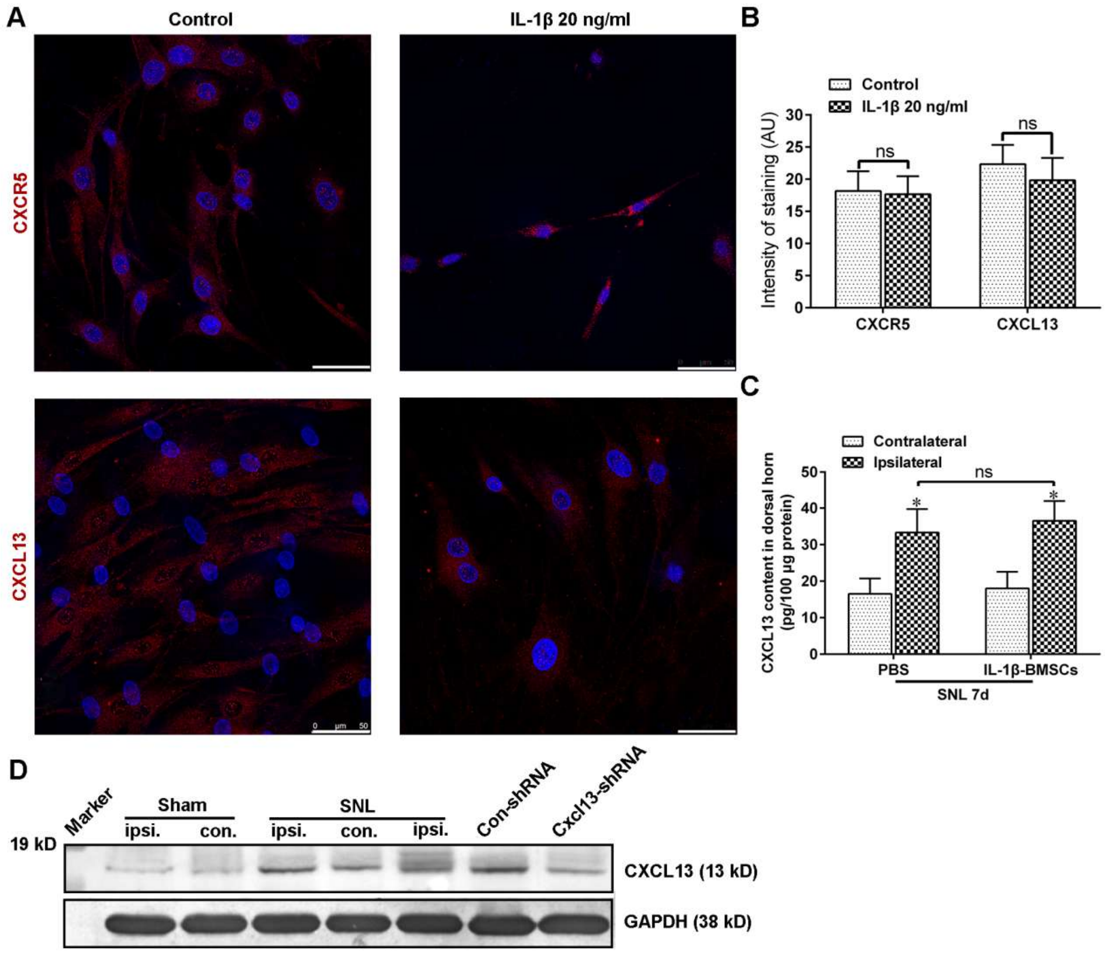


**Supplementary Figure S1.** (**A** ) Representative immunofluorescent images of CXCR5 and CXCL13 in BMSCs after IL-1β treatment. Scale bars = 50 μm. (**B**) Statistical results of intensity of staining in (**A**). (**C**) CXCL13 level in spinal cord on day 7 after IL-1β-BMSCs injection (SNL). (**D**) Expression of CXCL13 in spinal cords seven days after SNL or sham treatment. Control or Cxcl13 shRNA lentivirus vectors were injected three days before SNL. Statistical significance was determined by the student's t test for (**B**), or two-way ANOVA followed by Bonferroni's post-hoc test for (**C**). All data were expressed as the mean ± SEM.

## Supplementary Table S1. qRT-PCR Primer Sequences

| GENE | FORWARD (5′3′) | REVERSE (5′-3′) | ACCESSION |
|---|---|---|---|
| **Il-1β** | AGAGTGTGGATCCCAAACAA | AGTCAACTATGTCCCGACCA | NM_031512 |
| **Tnf-α** | CCAGGAGAAAGTCAGCCTCCT | TCATACCAGGGCTTGAGCTCA | NM_012675 |
| **Il-18** | CAGACCACTTTGGCAGACTT | ATCCTTCCATCCTTCACAG | NM_019165 |

## Supplementary Table S2. Target sequences of the shRNAs

| Sequence name | Sequence | Size | Accession |
|---|---|---|---|
| **Cxcl13 shRNA-1** | 5′-CAA ACA CGG CTG TAG ATT AAA-3′ | 21bp | NM_001017494 |
| **Cxcl13 shRNA-2** | 5′-CCT AGA CCC TAG AGG AAT AAA -3′ | 21 bp | |
| **Cxcl13 shRNA-3** | 5′- CAT CCA GTG TCA TTC AAT AAA-3′ | 21 bp | |
| **Control shRNA** | 5′-TTC TCC GAA CGT GTC ACG T-3′ | 19 bp | |
